# Transcriptome analysis reveals the key role of overdominant expression of photosynthetic and respiration-related genes in the formation of tobacco(*Nicotiana tabacum* L.) biomass heterosis

**DOI:** 10.1186/s12864-024-10507-8

**Published:** 2024-06-14

**Authors:** Anbin Lu, Shuaibo Zeng, Kai Pi, Benshan Long, Zejun Mo, Renxiang Liu

**Affiliations:** 1 College of Tobacco Science, Guizhou University, Guiyang, China; 2 College of Agriculture, Guizhou University, Guiyang, China; 3 Key Laboratory of Tobacco Quality in Guizhou Province, Guiyang, China

**Keywords:** Heterosis, Tobacco leaves biomass, Photosynthesis, Respiration.

## Abstract

**Background:**

Leaves are the nutritional and economic organs of tobacco, and their biomass directly affects tobacco yield and the economic benefits of farmers. In the early stage, our research found that tobacco hybrids have more leaves and larger leaf areas, but the performance and formation reasons of biomass heterosis are not yet clear.

**Results:**

This study selected 5 parents with significant differences in tobacco biomass and paired them with hybrid varieties. It was found that tobacco hybrid varieties have a common biomass heterosis, and 45 days after transplantation is the key period for the formation of tobacco biomass heterosis; By analyzing the biomass heterosis of hybrids, Va116×GDH94 and its parents were selected for transcriptome analysis. 76.69% of the differentially expressed genes between Va116×GDH94 and its parents showed overdominant expression pattern, and these overdominant expression genes were significantly enriched in the biological processes of photosynthesis and TCA cycle; During the process of photosynthesis, the overdominant up-regulation of genes such as *Lhc*, *Psa*, and *rbcl* promotes the progress of photosynthesis, thereby increasing the accumulation of tobacco biomass; During the respiratory process, genes such as *MDH*, *ACO*, and *OGDH* are overedominantly down-regulated, inhibiting the TCA cycle and reducing substrate consumption in hybrid offspring; The photosynthetic characteristics of the hybrid and its parents were measured, and the net photosynthetic capacity of the hybrid was significantly higher than that of the parents.

**Conclusion:**

These results indicate that the overdominant expression effect of differentially expressed genes in Va116×GDH94 and its parents plays a crucial role in the formation of tobacco biomass heterosis. The overdominant expression of genes related to photosynthesis and respiration enhances the photosynthetic ability of Va116×GDH94, reduces respiratory consumption, promotes the increase of biomass, and exhibits obvious heterosis.

**Supplementary Information:**

The online version contains supplementary material available at 10.1186/s12864-024-10507-8.

## Introduction

Heterosis refers to the phenomenon in which a hybrid is superior to its parents in various traits such as yield, quality, and resistance [1]. It has been widely used in various crops such as maize [2–4], rice [5, 6], rapeseed [7], and has made significant contributions to the improvement of crop yield. Heterosis theories and hypotheses about its genetic basis are constantly being proposed and supplemented. Currently, the dominance, overdominance and the epistasis are widely accepted and recognized. The dominance suggests that heterosis is controlled by multiple genes with explicit and implicit relationships, with dominance being advantageous while recessiveness is harmful. Hybrid F_1_ aggregates more dominant genes from both parents, thus exhibiting heterosis [8, 9]. The overdominance suggests that there is no overt recessive relationship between alleles from both parents, but there is interaction between them. In heterozygous conditions, the effect of allele interaction between parents is greater than that of homozygous genes [10]. The epistasis refers to the interaction between non allelic genes. The interaction between non allelic genes in hybrids results in hybrids exhibiting better performance than their parents [11–13]. With the development of quantitative genetics, the proposed “Hardy-Weinberg equilivbrium” [14, 15]. “Specific DNA methylation reprogramming pattern” [16]. and “Single-parent expression complementation model” [17] have promoted in-depth research on crop heterosis. Although these hypotheses explain its genetic basis in some biological traits [18]. the genetic mechanisms underlying heterosis formation in different crop traits are different, and theoretical research on heterosis formation still lags far behind practical applications.

With the rapid development of large-scale sequencing technology, RNA-seq provides an opportunity to study heterosis from the transcriptional level [19]. In rice [20, 21] and maize [22] studies, transcriptome results showed that differential gene expression between hybrid offspring and parents may contribute to plant heterosis. Through in-depth research, it was found that differentially expressed genes may be involved in important biological pathways for heterosis formation [23]. For example, based on RNA-seq technology, the molecular mechanism of maize ear heterosis was analyzed, and it was found that heterosis genes were mainly enriched in carbohydrate and protein metabolism processes, nitrogen assimilation and auxin metabolism pathways [24]. In rapeseed, previous studies [25] used RNA-seq technology to find that differential expression of genes related to plant hormone signaling and photosynthesis pathways jointly regulated the production of seedling heterosis in hybrids. In summary, changes in gene expression may alter biological regulatory networks, thereby affecting heterosis.

Photosynthesis is a vital biological process that enhances crop productivity [26]. Hybrids of various crops, such as Arabidopsis [27], maize [28], rapeseed [29], and rice [30, 31], have been shown to exhibit higher photosynthetic performance than their parents. This is attributed to the up-regulation of genes involved in photosynthesis, which boosts the light-harvesting capacity of crops [32], as well as the efficiency and transfer of solar radiation [33]. These factors promote chlorophyll synthesis, photosynthetic rate, and yield in leaves [34]. In contrast, substrate loss through respiration, which is negatively correlated with biomass accumulation [35], is reduced by the modulation of the tricarboxylic acid (TCA) cycle in hybrids [36]. Previous studies have reported that lowering the levels of TCA cycle intermediates can increase photosynthesis and growth rate in the whole carbon flux, leading to enhanced biomass [37]. However, further research is needed to elucidate the mechanisms of heterosis and plant growth regulation [38]. We propose that the enhancement of photosynthesis and the weakening of respiration may be one of the reasons for the emergence of biomass heterosis in hybrids.

*Nicotiana tabacum* L. is an economically important crop grown for its leaves. It has been widely used as a model plant to investigate various biological processes, owing to its high biomass, short life cycle, clear genetic background, ease of cultivation and genetic transformation [39–41]. Leaves are the main source of nutrients and harvestable biomass in tobacco, and they determine the economic value of the crop [42]. We previously found significant heterosis in leaf-related traits, such as leaf number [43, 44], upper leaf area [45], vein ratio of leaves [46], K + content [40, 47] and nicotine [48], with different genetic mechanisms underlying different traits. However, the expression and genetic basis of biomass heterosis in tobacco leaves remain unknown. Therefore, we chose parents with significant differences in biomass in tobacco leaves, combined with hybrids, and determined the biomass of hybrids at different stages to analyze the performance of biomass heterosis and clarify the key period for the formation of biomass heterosis; Using transcriptome analysis to identify genes and their expression patterns related to the formation of biomass heterosis in tobacco leaves, in order to clarify the physiological and molecular basis for the formation of biomass heterosis in hybrid varieties and provide a basis for the breeding of high-yield hybrid varieties.

## Results

### Tobacco leaf biomass and its heterosis performance

**Fig. 1:**
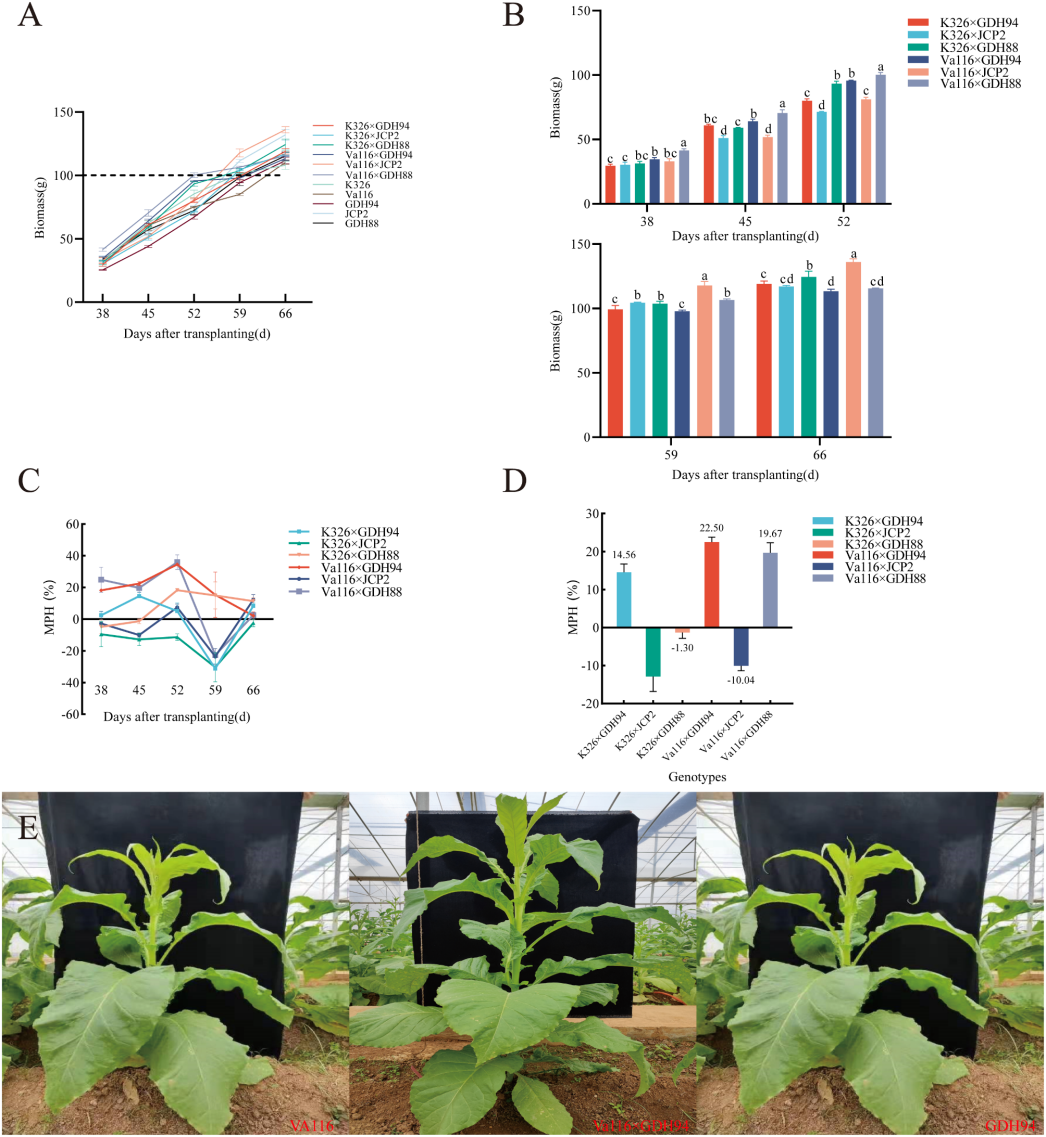
Biomass and heterosis values of the test materials **A** The trend of biomass changes in parents and hybrid varieties from 38 to 66 days after transplantation. **B** Differences in biomass of hybrid species from 38 to 66 days after transplantation. **C** The variation trend of hybrid biomass heterosis values from 38 days to 66 days after transplantation. **D** Comparison of Heterosis Values in Biomass of Hybrid Tobacco Leaves 45 Days After Transplantation. **E** Phenotypic images of VG9, Va116×GDH94 and GDH94. *Note* The average value of three biological replicates per material was used for mapping. The error bars represents the standard deviation of three biological replicates of biomass data for the same tobacco variety (line), indicating the degree of data dispersion. Significant differences of biomass at *p* < 0.05 were determined using Duncan’s new multiple range tests. The lowercase alphabets represent a significant difference (*p* < 0.05)

We investigated the biomass of parental and hybrid tobacco leaves at different growth stages (Fig. 1A) and found significant differences in biomass among different varieties (Supplementary file 1, Table 2; Fig. 1B). After 45–52 days of transplantation, the hybrid advantage of biomass reached 14.99% points, decreased by 12.90% points from 52 to 59 days after transplantation, and increased by 5.68% points from 59 to 66 days. This indicates that 45 to 52 days after transplantation is a critical period for the growth of tobacco biomass heterosis, and the growth trend has reached its peak (Fig. 1C). Therefore, 45 days after transplantation is a critical period for the formation of biomass heterosis in tobacco leaves. In order to explore the mechanism of biomass heterosis in tobacco leaves, we compared the heterosis values of different hybrids 45 days after transplantation (Fig. 1D) and found that VG9 (Va116×GDH94) had the highest heterosis (22.50%). Through phenotype analysis (Fig. 1E), it can be observed that VG9 has more leaves and a larger leaf area compared to its parents; Therefore, we conducted further transcriptome sequencing on Va116×GDH94, Va116, and GDH94.

### Analysis of transcriptome expression differences between hybrid combinations and their parents

To unravel the molecular mechanisms underlying the superior biomass performance of the hybrid Va116×GDH94, we performed RNA sequencing of biological samples collected 45 days after transplanting, using the Illumina HiSeq2000 platform (Supplementary file 1, Tables 3 and 4).

**Fig. 2:**
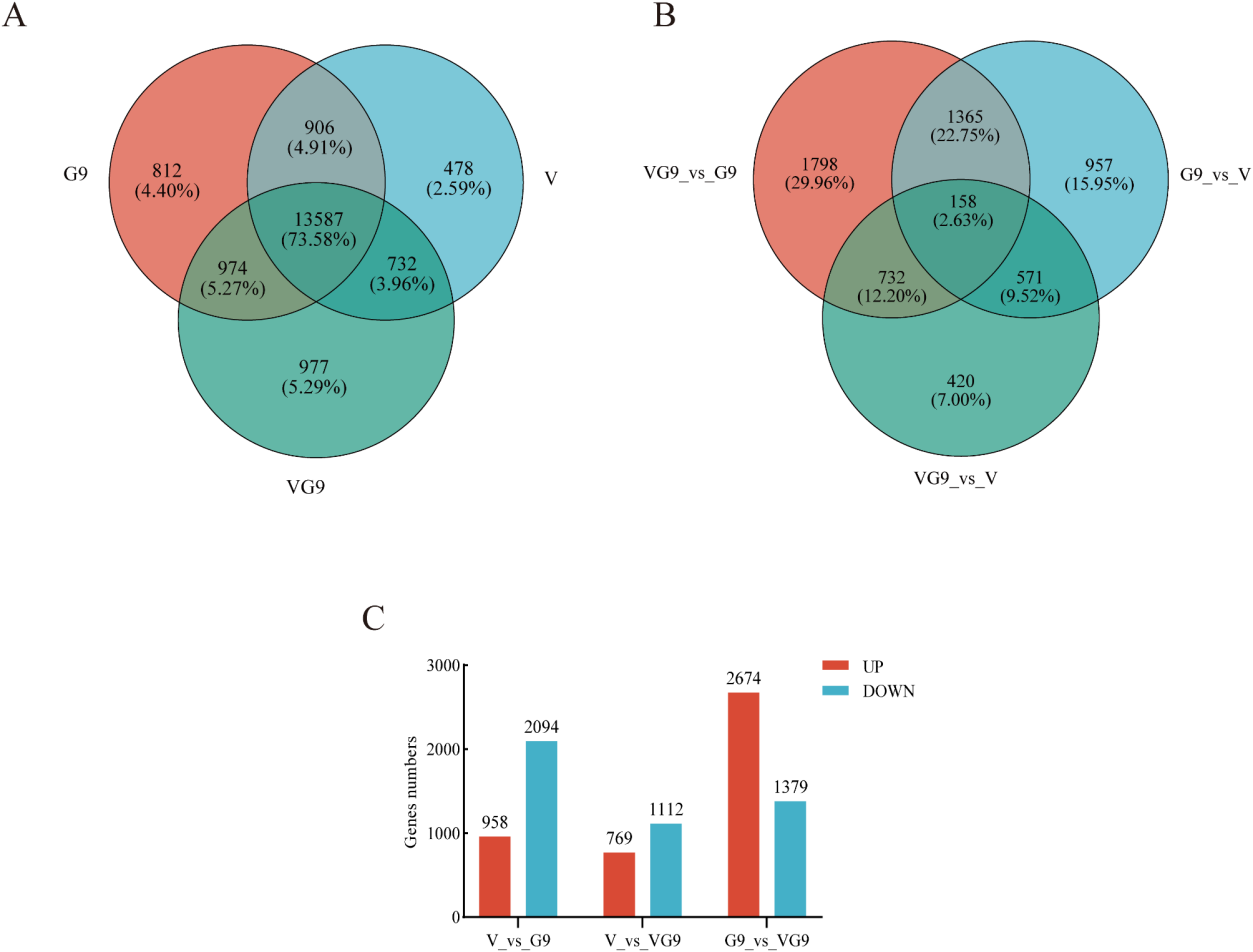
Statistical results of transcriptional profiles of different experimental materials and comparison groups. **A** Venn diagram shows the common and unique genes among the three genotypes of Va116, GDH94 and Va116×GDH94. **B** Venn diagram shows the common and unique genes in the three comparative groups of Va116×GDH94 vs. Va116, Va116×GDH94 vs. GDH94, and Va116 vs. GDH94. **C** Statistical analysis of differentially expressed genes upregulated and downregulated in three comparative groups. The horizontal axis represents different comparison groups, while the vertical axis represents the corresponding number of up and down regulated genes. Red represents up regulation, blue represents down regulation *Note* V: Va116, G: GDH94, VG9: Va116×GDH94. The screening criteria for significantly differentially expressed genes are: *p* < 0.05&|log2FC|≥2. When a gene meets both conditions, it is considered as a differentially expressed gene (DEG)

To identify genes and pathways responsible for the heterosis advantage in tobacco, we compared the transcriptional profiles of Va116×GDH94 and its parents Va116 and GDH94. We focused on the 13,587 genes that were simultaneously identified in the three genotypes (Fig. 2A). At significant levels *p* ≤ 0.05 and |log2 Fold Change| ≥ 2, we identified 1112 up-regulated and 769 down-regulated genes between Va116×GDH94 and Va116, 1,379 up-regulated and 2,674 down-regulated genes between Va116×GDH94 and GDH94 (Supplementary file 2, Sheet 1, 2, Fig. 2C). Venn diagram analysis of the differentially expressed genes (DEGs) between the comparison groups (Fig. 2B) showed that VvsG9, VG9vsV, and VG9vsG9 had 957, 420, and 1798 unique DEGs, respectively, with only 158 shared DEGs in the three comparison groups (Fig. 2B). These results suggest that the transcriptional profiles of the three genotypic materials are significantly different and that the DEGs may be responsible for the heterosis advantage of the tobacco cross combinations.

### Identification and analysis of differential gene expression patterns in hybrids

**Fig. 3:**
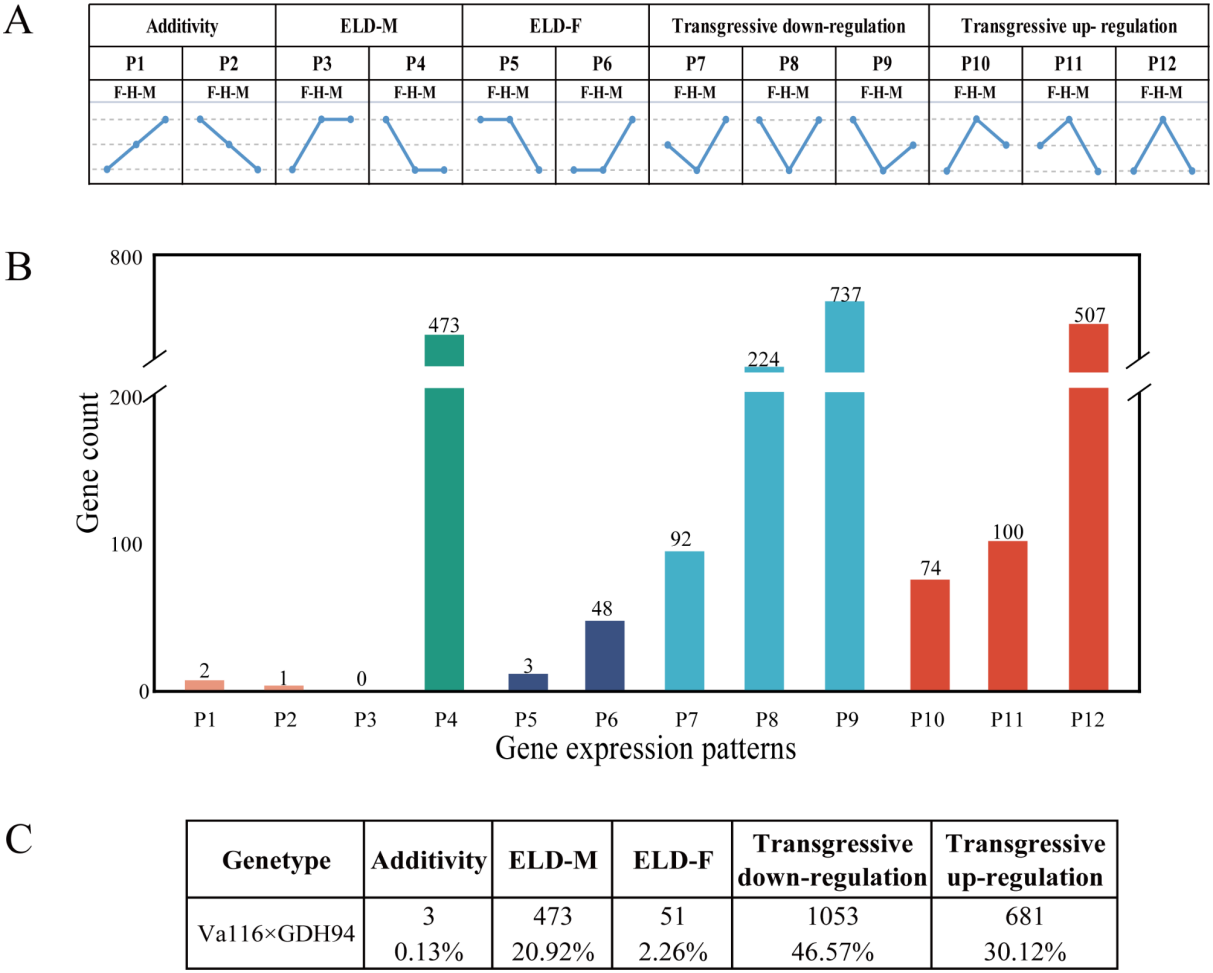
Identification and classification of differential gene expression patterns in hybrids. **A** Classification of the 12 expression patterns. Genes in P1 and P2 patterns exhibited additive expression. Genes in P3–P6 patterns were the dominant expression. Genes in the P7-P12 pattern exhibited a transgressive expression, among which P7–P9 were down-regulated overdominant expression, whereas P10–P12 genes were up-regulated overdominance. **B** The distribution of differentially expressed genes in hybrid species among 12 expression patterns. **C** The number and proportion of DEGs in the five expression patterns

In order to gain further insight into the potential role of these DEGs in the formation of tobacco biomass heterosis, we classified the differentially expressed genes into 12 expression patterns (P1-P12) (Supplementary file 2, Sheet 3) to examine the contribution of additive and non-additive gene expression patterns to tobacco biomass heterosis. We grouped these 12 expression patterns into five broad categories (Fig. 3A). Interestingly, only 0.13% of the genes in the hybrid Va116×GDH94 exhibited an additive expression pattern between the two parents (P1 and P2) (Fig. 3B and C), indicating that the formation of biomass heterosis in tobacco is largely influenced by the non-additive effect of genes. Among the non-additive DEGs, 23.18% (P3-P6) displayed a dominant expression pattern, while 76.69% (P7-P12) showed overdominant expression pattern, suggesting that the overdominant effect of gene expression levels may play a major role in conferring the heterosis in tobacco leaves biomass.

### Functional enrichment analysis of overdominant genes

**Fig. 4:**
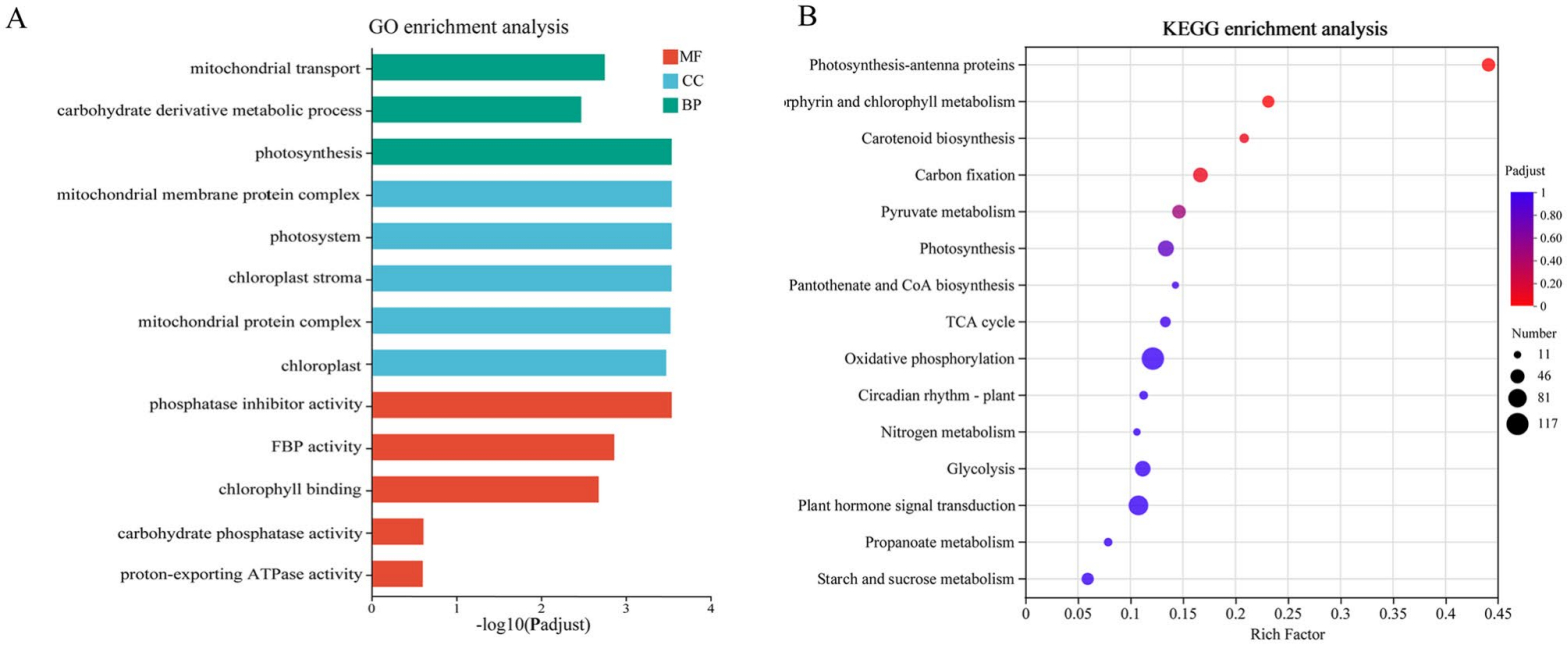
Functional enrichment analysis of overdominant expression pattern genes. **A** GO enrichment analysis of overdominant expression pattern genes, where the vertical axis represents GO term and the horizontal axis represents the significance level of enrichment, corresponding to the height of the column. The smaller the FDR, the greater the - log10 (Padjust) value, and the more significantly enriched the GO term. The three colors represent three major classifications, namely biological processes (BP), cellular components (CC), and molecular functions (MF). **B** KEGG enrichment analysis of overdominant expression pattern genes. The vertical axis represents the name of the pathway, and the horizontal axis represents the ratio of the number of enriched genes/transcripts (Sample number) in the pathway to the number of annotated genes/transcripts (Background number). The larger the Rich factor, the greater the degree of enrichment

To explore the biological functions of the overdominant genes in the hybrids, GO enrichment analysis was first performed on the overdominant genes in the hybrid (Fig. 4A, Supplementary file 2, Sheet 4). The results showed that the overdominant genes were mainly enriched in molecular functions such as chlorophyll binding, carbohydrate phosphatase activity, and phosphatase inhibitor activity. They were also significantly enriched in cellular components such as chloroplast, mitochondrial protein complex, and photosystem. Analysis of biological processes showed that these genes were significantly enriched in carbohydrate derivative metabolic processes, photosynthesis, and mitochondrial transport.

KEGG enrichment analysis (Fig. 4B, Supplementary file 2, Sheet 5) showed that these overdominant genes were mainly enriched in biological pathways such as photosynthesis antenna proteins, the TCA cycle, and carbon fixation in photosynthetic organisms (Fig. 4B). The results suggested that the overdominance expression of genes might enhance or inhibit the photosynthetic antenna protein, the carbon fixation process of photosynthetic organisms, and the tricarboxylic acid cycle process, which could lead to the formation of heterosis in tobacco biomass.

### Up-regulation of overdominant photosynthesis related genes promotes the formation of tobacco biomass

**Fig. 5:**
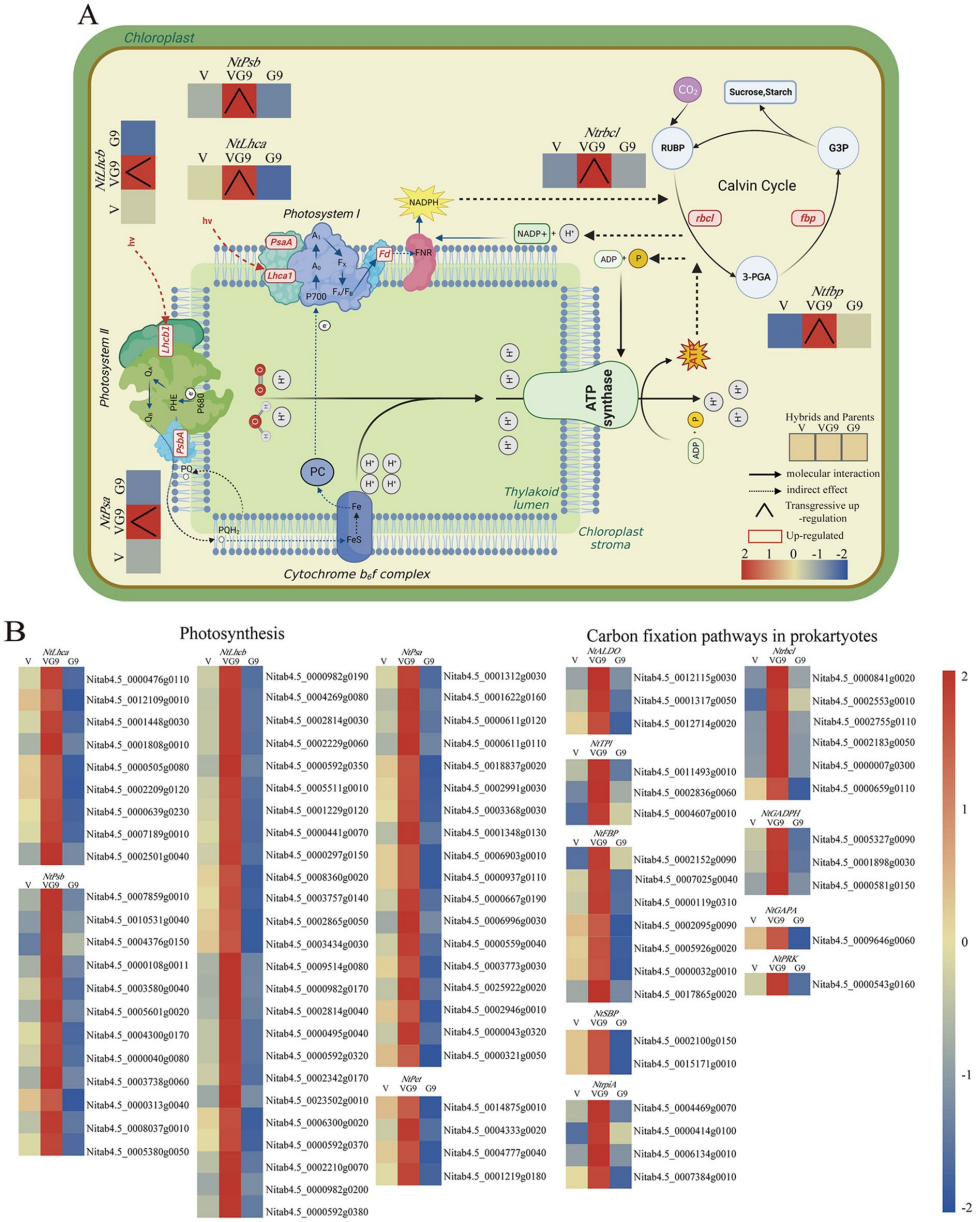
Photosynthesis of hybrid **A** Distribution of overdominant expression genes in photosynthesis related pathways in Va116×GDH94. Up-regulated genes are represented by a red box, while overdominant expression pattern is represented by a regular triangle. **B** Heat map of gene expression levels of overdominant expression pattern in photosynthesis related pathways of hybrid. *Note Lhca*: Light-harvesting complex I chlorophyll a/b binding protein 1. *Lhcb*: Light-harvesting complex II chlorophyll a/b binding protein 1. *Psb*: Photosystem II P680 reaction center D1 protein. *Psa*: Photosystem I P700 chlorophyll a apoprotein. *PetF*: Ferredoxin. *RipA*: Ribose 5-phosphate isomerase A. *PRK*: Phosphoribulokinase. *rbcl*: Ribulose-bisphosphate carboxylase large chain. *GAPDH*: Glyceraldehyde 3-phosphate dehydrogenase (phosphorylating). *GAPA*: Glyceraldehyde-3-phosphate dehydrogenase (NADP+). *TPI*: Triosephosphate isomerase (TIM). *ALDO*: Fructose-bisphosphate aldolase, classI. *FBP*: Fructose-bisphosphate aldolase, class I. *MDH*: malate dehydrogenase. *SBP*: Sedoheptulose-bisphosphatase

By analyzing the biological processes of the overdominant expression genes of the hybrid Va116×GDH94, we found that the overdominant expression genes were significantly enriched in photosynthesis and carbon fixation in photosynthetic organisms. In this study, we identified 98 genes in the hybrid that showed up-regulated overdominant expression pattern (Fig. 5B). During the light harvesting process, 36 genes encoding Lhc protein in the hybrid exhibited overdominant up-regulation expression; 18 and 12 genes encoding Psa and Psb in Photosystem I and Photosystem II, respectively, also showed overdominant up-regulation expression. These genes play important roles in light energy transfer and protection, which are beneficial for the photosynthesis of the hybrid. There were 5 genes encoding ATP synthesis that showed overdominant up-regulation, which can promote the reduction of inorganic CO_2_ to organic carbon. In the carbon fixation in photosynthetic organisms, six rbcl genes showed overdominant up-regulation expression pattern. These results indicate that the genes that are overdominantly up-regulated during the photoresponse stage may lead to more excitation energy being transferred from the antenna protein of the photosystem to the photosynthetic reaction center. The increase of ATP can reduce more inorganic CO_2_ to organic carbon in the Calvin cycle, and directly affect the photosynthetic rate and carbohydrate accumulation of the hybrid combinations (Fig. 5A). The results indicate that the overdominant up-regulation of genes related to light response and carbon fixation in photosynthetic organisms pathways in the hybrid is an important reason for the formation of biomass heterosis in tobacco leaves.

### The regulation of tobacco biomass consumption by genes related to the tricarboxylic acid cycle pathway

**Fig. 6:**
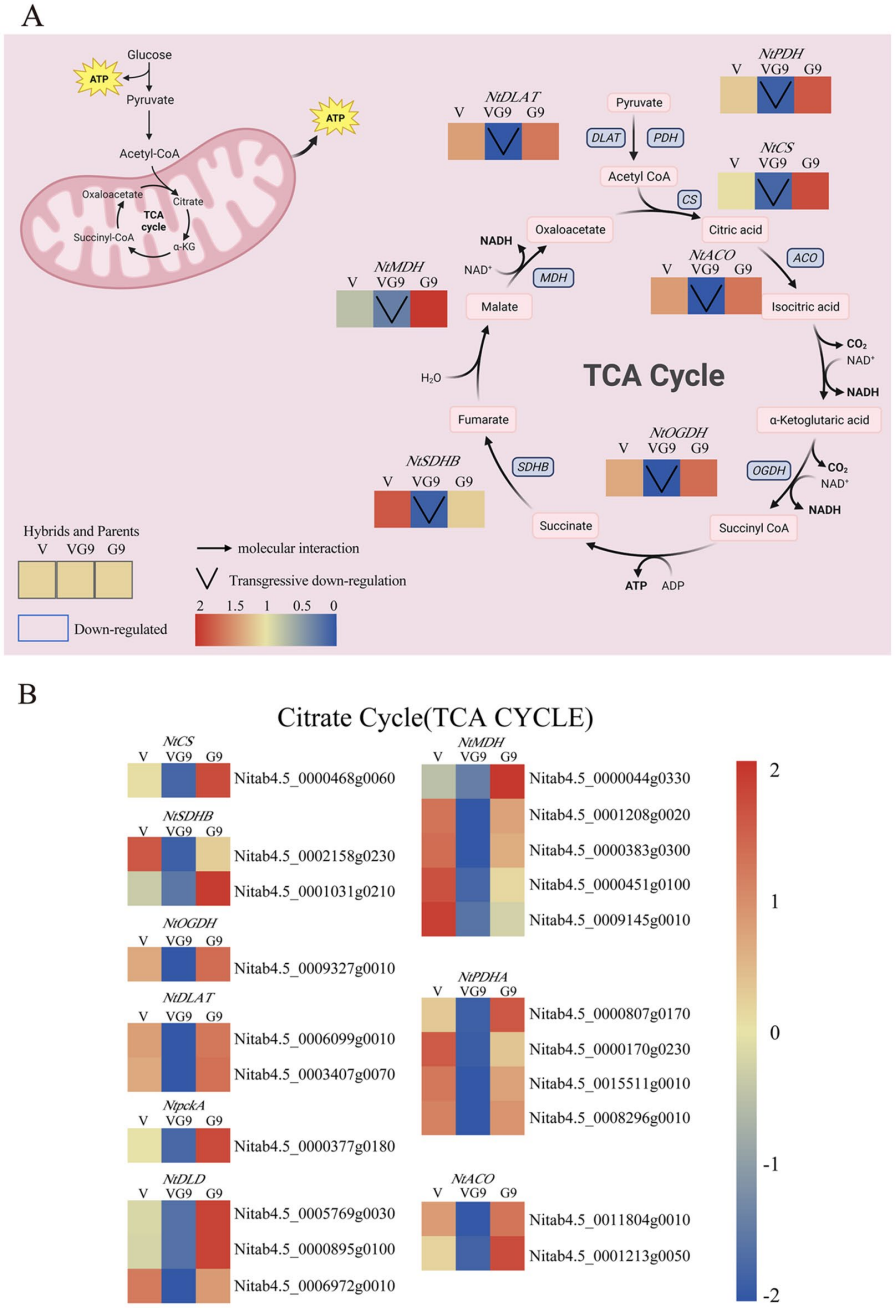
Respiratory function of hybrid (Tricarboxylic acid cycle) **A** Distribution of overdominant expression pattern genes in the tricarboxylic acid cycle pathway of Va116×GDH94, with down-regulated genes represented by blue boxes and overdominant expression pattern represented by inverted triangles. **B** Heat map of gene expression levels of overdominant expression pattern in the tricarboxylic acid cycle pathway of hybrid. *Note CS*: Citrate synthase. *MDH*: Malate dehydrogenase. *SDHB*: Succinate dehydrogenase (ubiquinone) iron-sulfur subunit. *DLD*: Succinate dehydrogenase (ubiquinone) iron-sulfur subunit. *OGDH*: 2-Oxoglutarate dehydrogenase E1 component. *ACO*: Aconitate hydratase. *PDHA*: Pyruvate dehydrogenase E1 component subunit alpha. *DLAT*: Pyruvate dehydrogenase E2 component (dihydrolipoyllysine-residueacetyltransferase). *pckA*: Phosphoenolpyruvate carboxykinase

In the tricarboxylic acid cycle, we identified 21 genes in the hybrid that showed down-regulated overdominant expression pattern (Fig. 6B) in this study. During the conversion of pyruvate to acetyl CoA, 4 *PDHA* and 2 *DLAT* were overdominantly down-regulated, inhibiting the synthesis of acetyl CoA. During the conversion of acetyl CoA to citrate, 1 *CS* was overdominantly down-regulated; during the conversion to isocitrate, 2 *ACO* were overdominantly down-regulated. During the conversion of α-ketoglutarate to succinyl CoA, 1 *OGDH* was overdominantly down-regulated; during the conversion from succinate to malate, 2 *SDHB* were overdominantly down-regulated. During the conversion from malate to oxaloacetate, 5 *MDH* were overdominantly down-regulated, inhibiting the tricarboxylic acid cycle and reducing the consumption of respiratory substrates (Fig. 6A). The results showed that the hybrid had lower respiratory expenditure compared to their parents, promoting the formation of biomass heterosis in tobacco leaves.

### Enhanced photosynthetic capacity of hybrids

**Fig. 7:**
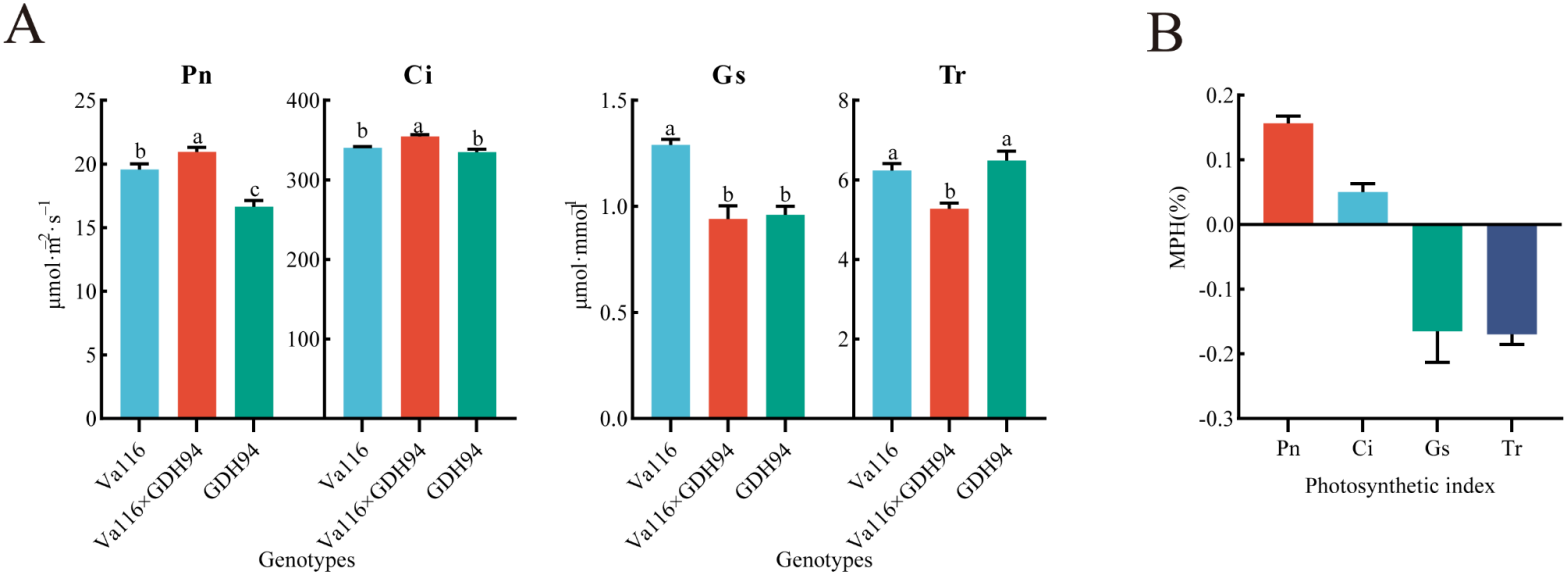
Measurement of photosynthetic parameters in hybrid and its parents. **A** P_n_, C_i_, G_s_ and T_r_ of the hybrid and its parents. **B** Heterosis values for P_n_, C_i_, G_s_ and T_r_ in the hybrid

The results of the measurement of photosynthetic parameters of hybrid and its parents (Fig. 7, Supplementary file 1, Table 5) suggest that the overdominance effect of photosynthesis-related genes in the hybrid contributes to the formation of heterosis in tobacco biomass. Specifically, the hybrid showed significantly higher P_n_ and C_i_, which are positive dominant values, indicating a stronger photosynthetic capacity compared to its parents. On the other hand, the hybrid showed significantly lower Tr, which is a negative dominant value, indicating less transpiration consumption. However, the Gs of Va116 was significantly higher than Va116×GDH94 and GDH94, suggesting that the hybrid may have inherited the trait of lower Gs from GDH94. Overall, these results suggest that the overdominance effect of photosynthesis-related genes in the hybrid promoted photosynthesis and reduced transpiration, leading to the formation of heterosis in tobacco biomass.

### qRT-PCR analysis

**Fig. 8:**
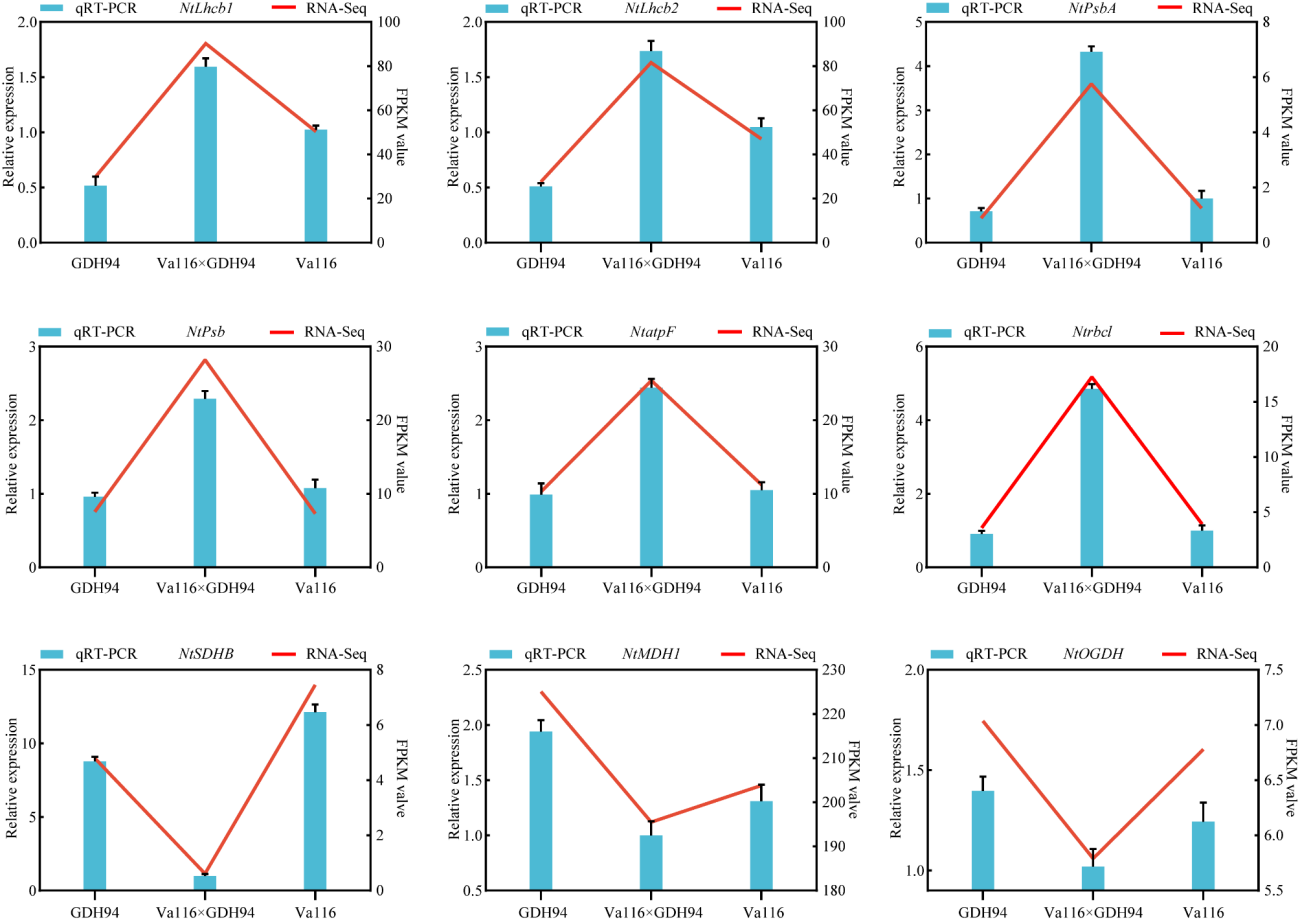
Comparison of photosynthesis and respiration related gene expression levels of RNA-seq and qRT-PCR in hybrid and its parents

We focused on six genes related to photosynthesis and three genes related to respiration, and examined their expression levels in the hybrid Va116×GDH94 using qRT-PCR (Fig. 8). The results showed that all six genes related to photosynthesis showed up-regulated overdominant expression pattern, while three genes related to respiration showed down-regulated overdominant expression pattern, indicating that the overdominant expression pattern of genes in hybrid varieties may be the key to the formation of biomass heterosis in tobacco leaves.This finding sheds light on the genetic mechanisms underlying the observed heterosis and provides important insights for future breeding strategies aimed at improving crop yields.

## Discussion

Heterosis is a natural phenomenon in which hybrid offspring show traits that surpass their parents in terms of yield, adaptability, and response to biotic and abiotic stresses [49]. Due to the significant improvement in crop yield and resistance caused by heterosis, it has been widely used in agricultural production [50]. As an economic crop, tobacco leaves biomass directly affects yield and the economic benefits of farmers. This study found that tobacco leaves biomass has significant heterosis. By selecting suitable parents and using heterosis utilization methods, hybrids with suitable biomass can be bred. In the study of the formation mechanism of heterosis in different crops, previous studies [50] confirmed the dominance hypothesis in rice and also provided guidance for the aggregation of dominant loci; multiple reports have shown that epistasis plays an important role in the regulation of heterosis in different traits in rice [51] and maize [52]; at present, there have been multiple reports proving the existence of overdominance: *AtFT* encodes a floral factor and participates in the regulation of flowering time in Arabidopsis. Its homologous gene *OsHd3a* in rice [50] and homologous gene *SlSFT* in tomato [53] both show strong overdominant effects in yield heterosis. This study performed transcriptome analysis on hybrid Va116×GDH94 and found that 76.69% of DEGs mainly showed overdominant expression pattern, indicating that the overdominant effect of gene expression levels may play a major role in the formation of biomass heterosis in tobacco leaves, consistent with the research results of Hayford [54] on cotton biomass heterosis.

Photosynthesis is closely related to biomass heterosis[3, 18, 25,], the key process for crop biomass formation [55]. Improving light-harvesting ability is a prerequisite for optimizing photosynthesis. The light-harvesting chlorophyll a/b binding proteins, comprising two evolutionary groups, Lhca and Lhcb [56], act as the main external antenna around the reaction center of PS II [32]. They quickly transmit the received light energy to the reaction center, involved in light energy conversion and transfer [33, 57], and play an important role in the photosynthetic system [58, 59]. We find that *Lhc* in Va116×GDH94 shows overdominant expression pattern, which improves the light-harvesting ability of Va116×GDH94, resulting in a higher net photosynthetic rate than the parents, thus showing heterosis in tobacco biomass. This is consistent with previous research results [60]. Va116×GDH94 encoding PsbA (D1) and CP43 in Photosystem II show overdominant up-regulation of genes in Photosystem I such as *Psa*, *Psb*, and chloroplast ATP synthase, suggesting improved plant abilities in charge separation, charge stability, photoprotection, stress resistance [61–63] and ATP synthesis, further driving the Calvin-Benson-Bassham metabolic cycle [64]. The overdominant expression of the *rbcl* further promotes the conversion of CO_2_ into biomass. Our research results indicate that overexpression of photosynthesis related genes in the hybrid combination Va116×GDH94 promotes the photosynthesis and carbon sequestration pathways of photosynthetic organisms, thereby forming heterosis in tobacco biomass.

Nearly half of the carbon fixed by plants through photosynthesis is returned to the atmosphere through their respiration [65]. Reducing plant respiration may therefore increase crop yield [66]. Previous studies have shown that respiration consumption decreases rice yield [67], mainly because most of the energy generated by respiration is allocated to maintenance rather than growth respiration. Plant respiration is thus one of the main causes of yield loss [68]. Compared with its parents, hybrid larch has higher photosynthetic efficiency and lower respiration rate, providing more carbon-based resources for wood production [69]. The TCA cycle consists of eight enzymes: citrate synthase (CSY), aconitase (ACO), isocitrate dehydrogenase (IDH), α-ketoglutarate dehydrogenase complex (OGDH), succinyl CoA synthase (SCoAL), succinate dehydrogenase (SDH), fumarate dehydrogenase (FUM), and malate dehydrogenase (MDH) [70]. This study identified overexpression of *MDH*, *ACO*, *FUM*, *OGDH*, and *SDH* in the TCA cycle of Va116×GDH94. Previous studies have shown that down-regulation of these genes affects respiration, increases photosynthetic rate, and biomass of tomatoes, potatoes, and cherries [71–75]. Previous studies have found that overexpression of *AtLPD* in Arabidopsis promotes photosynthesis and TCA cycle, but ultimately leads to biomass accumulation, which is inconsistent with the results of this study. We speculate that higher GDC reduces carbon flow in the photorespiration pathway and decreases the accumulation of photorespiring metabolites in Va116×GDH94. At present, most research on crop heterosis focuses on accumulation, while there is relatively little research on consumption analysis, which may be the direction we need to pay attention to in the future.

Leaves are the nutritional and economic organs of tobacco, and their biomass directly affects tobacco yield and the economic benefits of tobacco farmers. Previous research found that tobacco hybrids have more and larger leaves [44], but the performance and formation mechanisms of biomass heterosis in tobacco leaves are not clear. Here, we analyzed the key period for the formation of biomass heterosis in tobacco leaves, selected the hybrid Va116×GDH94 with the highest heterosis value from the test materials, and used transcriptome technology to investigate the causes of biomass heterosis in tobacco leaves. We found that the overdominant effect of gene expression level may play a major role in the formation of biomass heterosis in tobacco leaves. We analyzed the pathways of photosynthesis and respiration that were enriched, and found that genes with overdominant up-regulation expression in the photosynthesis pathway improved tobacco’s light capture efficiency and light protection ability, enhanced carbohydrate accumulation, while genes with overdominant down-regulation expression in the respiration pathway reduced substrate consumption by respiration, thereby promoting the formation of biomass heterosis in tobacco leaves.

## Conclusion

This study indicates that tobacco biomass exhibits a universal heterosis. The transcriptome analysis results showed that the overdominant effect of differentially expressed genes plays an important role in the formation of biomass heterosis in Va116×GDH94. The biomass heterosis of Va116×GDH94 can be attributed to the overdominant up-regulation of genes in the photosynthetic pathway, such as *NtLhc*, *NtPsa*, *Ntrbcl*, as well as the overdominant down-regulation of genes in the respiratory pathway, such as *NtSDHB*, *NtDLAT*, and *NtMDH*. This study provides new insights for revealing the biomass heterosis of tobacco leaves and theoretical support for tobacco hybrid breeding.

## Materials and methods

### Experimental materials

In this research, we selected five tobacco varieties (lines) with substantial differences in leaf biomass as parents, specifically K326 and Va116 as one parent, and GDH94, JCP2, and GDH88 as the other parents. Using an Appling incomplete diallel crossing design (NCII), we produced six hybrids with a total of 11 experimental materials. The seeds for each experiment were provided by the Key Laboratory of Tobacco Quality Research in Guizhou Province. We ensured that the collection of plant material and the experimental and field studies of the plants complied with the relevant institutional, national, and international guidelines and legislation. The seeds were sown in a greenhouse using floating seedlings and were transplanted to the field once the seedlings had grown to contain five true leaves. The field trial was carried out in a randomized group design with three biological replications in 2022 at the Guizhou University Tobacco Research Experimental Base, with a row-plant spacing of 110 × 55 cm. The field cultivation management measures were executed according to the Guizhou Standard Quality Tobacco Production Program.

### Determination of tobacco leaf biomass and analysis of hybrid advantage

From 38 to 66 days after transplanting, tobacco leaf biomass was measured at 7-day intervals for a total of 5 times. Three healthy and well-grown tobacco plants were selected from each plot, and 9–11 leaf positions were selected for mixing. Three replicates were taken. Fresh biological samples were frozen with liquid nitrogen and then stored in an ultra-low temperature refrigerator at -80℃. The biomass of tobacco leaves was determined using the method of drying and weighing. The whole plant leaves were harvested and heated at 105℃ for 30 min, and then dried to a constant weight at 75℃ to determine their dry weight. We ensured that the sample collection and handling procedures followed relevant institutional, national, and international guidelines and regulations.

The value of mid-parent heterosis were calculated according to the following method:

$$MPH(\% ) = ({{{F_1} - MP} \over {MP}}) \times 100$$

F_1_ indicates the value of hybrid, MP indicates the average value of both parents.

### Transcriptome sequencing and data alignment

RNA was extracted from leaf tissue using the TRIzol (Invitrogen) method. Total RNA was extracted from tissue using TRIzol® reagent (plant RNA purification reagent for plant tissue) according to the manufacturer’s instructions (Invitrogen), and genomic DNA was removed using DNase I (TaKara).The concentration and purity of the extracted RNA were measured by Nanodrop2000, The TruSeqTM RNA sample preparation Kit (Illumina, San Diego, CA) was used to build RNA libraries. High-throughput sequencing was performed using the NovaSeq 6000 sequencing platform with a read length of PE150.

Using software HiSat2 Integrate the raw data after quality control with the tobacco K326 reference genome Conduct a comparison [76] to obtain Reads for subsequent transcript assembly and expression calculation.

### Screening and functional analysis of differential genes

Quantitative analysis of gene expression levels used software RSEM [77]. Differential expression analysis was performed using the DESeq2 [78].The screening criteria for significantly differentially expressed genes are: *p* < 0.05 and | log2FC | ≥ 2. When a gene satisfies both of these conditions, it is considered a differentially expressed gene (DEG). Using STEM software and referring to the methods of Rapp [79] and Yoo [80], differentially expressed genes were classified into 12 expression patterns. GO functional enrichment was carried out by Goatools [81]. Using R software for KEGG pathway enrichment analysis, when the *P*-value ≤ 0.05, it is considered that there is significant enrichment in the KEGG pathway.

### qRT-PCR validation

To validate the accuracy of the transcriptome data, we selected six DEGs for qRT-PCR (PCR quantitative real-time) analysis. The SYBR Premix Ex Taq kit (Takara) and the Applied Biosystems 7500 real-time PCR system (Life Technologies Corporation, Beverly, MA, USA) were used to conduct the experiments. The genes and corresponding primers used for qPCR are listed in Supplementary file 1, Table 1. The relative expression of each gene was calculated using 2^−ΔΔCt^ [82].

### Measurement of photosynthetic indexes

Photosynthetic measurements were conducted using the LI-6400 portable photosynthetic analyzer manufactured by the LICOR company in the United States. The measurements were carried out in clear and windless weather conditions from 9:00 to 11:00 am, during the same period as the biomass measurements. The net photosynthetic rate (P_n_), transpiration rate (T_r_), intercellular CO_2_ concentration (C_i_), stomatal conductance (G_s_). Measured the 9–11 leaves and performed three biological replicates.

### Electronic supplementary material

Below is the link to the electronic supplementary material.

Supplementary Material 1

Supplementary Material 2

## Data Availability

Sequence data that support the findings of this study have been deposited in NCBI under GEO accession number GSE252097.Data is provided within the supplementary information files.

## Abbreviations

DEGs: Differentially expressed genes
TCA cycle: Tricarboxylic acid cycle
P_n_: Net photosynthetic rate
C_i_: Intercellular CO_2_ concentration
T_r_: Transpiration rate
G_s_: Stomatal conductance
