# Supplementary material for: Transcriptome analysis reveals the key role of overdominant expression of photosynthetic and respiration-related genes in the formation of tobacco(Nicotiana tabacum L.) biomass heterosis
Source: BMC Genomics. 2024 Jun 14;25:598. doi: 10.1186/s12864-024-10507-8 (PMC11177473; doi:10.1186/s12864-024-10507-8)
Supplement: Supplementary file 2 — Supplementary Material 2 [file 12864_2024_10507_MOESM2_ESM.doc]

Supplementary Material 1

**Transcriptome analysis reveals the key role of overdominant expression of photosynthetic and respiration-related genes in the formation of tobacco(*Nicotiana tabacum* L.) biomass heterosis**

**Anbin Lu^1,3^, Shuaibo Zeng^1,3^, Kai Pi^1,3^, Benshan Long^1,3^,Zejun Mo^2,3^, Renxiang Liu^1,3*^**

^1^College of Tobacco, Guizhou University, Guiyang, China

^2^College of Agriculture, Guizhou University, Guiyang, China

^3^Key Laboratory of Tobacco Quality in Guizhou Province, Guiyang, China

*** Correspondence:**

Corresponding Author: Renxiang Liu

rxliu@gzu.edu.cn

| **TABLE 1** │ Genes and corresponding primers used for qRT-PCR | | | |
| --- | --- | --- | --- |
| **Gene** | **sequence number** | **Primer sequence** | **annealing temperature(°C)** |
| *NtLhcb1* | Nitab4.5_0002814g0050 | F:TGGGACACTGCTGGACTTT | 58.3 |
|  |  | R:GCAACACGGTAACCCTCAA |  |
| *NtLhcb2* | Nitab4.5_0005511g0010 | F:TCTCCGAGCAAACTCCATC | 56.3 |
|  |  | R:GCCTCCGCCAACTCTGTAT |  |
| *NtPsbA* | Nitab4.5_0008665g0010 | F:TGCTGCTCCTCCAGTAGACA | 52.0 |
|  |  | R:AATCCAAGGTCGCATACCC |  |
| *NtPsb* | Nitab4.5_0003961g0020 | F:TATTGCCTTCATTGCTGCTC | 50.0 |
|  |  | R:TCATCAACGGATGCCACTT |  |
| *NtatpF* | Nitab4.5_0003634g0020 | F:ATCAGCAGCACTAAACAAG | 53.9 |
|  |  | R:TCATCACTAAGAGCAGCAA |  |
| *NtrbcL* | Nitab4.5_0000841g0020 | F:TATCGCCAACGAGAAATCG | 50.7 |
|  |  | R:CCTGAAGCCACGGGTAAAA |  |
| *NtSDHB* | Nitab4.5_0002158g0230 | F:TCTTATCTTGGTCCCGCTGC | 58.6 |
|  |  | R: GTCAAGGCGCTCCTGAGTAT |  |
| *NtMDH1* | Nitab4.5_0000044g0330 | F:TCACACCAAAACGGATTGTAACT | 59.7 |
|  |  | R:CGCCACTGTAGGACTAGGAA |  |
| *NtOGDH* | Nitab4.5_0009327g0010 | F:ACATGCAAAAATGGAGAATGGTGTG | 61.2 |
|  |  | R:CTCAGCTGTTGCGTCCAACTT |  |
| *NtLR25* | Nitab4.5_0002995g0050 | F:CCCCTCACCACAGAGTCTGCA | 62.0 |
|  |  | R: AAGGGTGTTGTTGTCCTCAATCTT |  |

| **TABLE 2** │ Analysis of differences in biomass between parental and hybrid tobacco leaves（g） | | | | | |
| --- | --- | --- | --- | --- | --- |
| **Genetype** | **DAT38** | **DAT45** | **DAT52** | **DAT59** | **DAT66** |
| K326 | 32.27bcde | 62.49bc | 85.45c | 101.52def | 108.07g |
| Va116 | 32.96bcd | 60.55bcd | 75.15ef | 85.00h | 110.34fg |
| GDH94 | 25.46f | 43.99g | 67.07h | 94.29g | 111.72efg |
| JCP2 | 34.77b | 54.64ef | 75.69e | 111.41b | 132.07a |
| GDH88 | 33.53bc | 57.12de | 72.32fg | 98.46fg | 115.35cde |
| K326×GDH94 | 29.58e | 60.99bcd | 80.03d | 99.33ef | 119.05c |
| K326×JCP2 | 30.29de | 51.03f | 71.42g | 104.41cd | 117.04cd |
| K326×GDH88 | 31.28cde | 59.02cd | 93.31b | 103.75cde | 124.47b |
| Va116×GDH94 | 34.51b | 64.01b | 95.68b | 97.83fg | 113.35def |
| Va116×JCP2 | 32.91bcd | 51.81f | 81.03d | 117.79a | 136.11a |
| Va116×GDH88 | 41.43a | 70.42a | 100.2367a | 106.52c | 115.55cde |

NOTE: Biomass measurement methods: The biomass of tobacco leaves was determined using the method of drying and weighing. The whole plant leaves were harvested and heated at 105 ℃ for 30 minutes, and then dried to a constant weight at 75 ℃ to determine their dry weight.The lowercase alphabets represent a significant difference (*p* < 0.05).

Unit: grams (g).

| **TABLE 3** │ Statistical table of sequencing data of three materials | | | | | | | | |
| --- | --- | --- | --- | --- | --- | --- | --- | --- |
| **Sample** | **Raw reads** | **Raw bases** | **Clean reads** | **Clean bases** | **Error rate(%)** | **Q20(%)** | **Q30(%)** | **GC content(%)** |
| G9_1 | 80982992 | 12228431792 | 79565184 | 11751652246 | 0.0265 | 97.53 | 92.77 | 43.27 |
| G9_2 | 80542130 | 12161861630 | 79232272 | 11705048014 | 0.0266 | 97.5 | 92.7 | 43.3 |
| G9_3 | 87523282 | 13216015582 | 86269428 | 12663615035 | 0.0263 | 97.59 | 92.95 | 43.31 |
| V_1 | 81538888 | 12312372088 | 80068028 | 11815692671 | 0.0266 | 97.47 | 92.7 | 43.37 |
| V_2 | 85913928 | 12973003128 | 84445126 | 12501624278 | 0.0264 | 97.54 | 92.83 | 43.33 |
| V_3 | 70044446 | 10576711346 | 69087002 | 10244803178 | 0.0261 | 97.7 | 93.12 | 43.25 |
| VG9_1 | 73741352 | 11134944152 | 71341098 | 10680849105 | 0.0265 | 97.53 | 92.64 | 42.77 |
| VG9_2 | 73264870 | 11062995370 | 70879396 | 10563117176 | 0.0272 | 97.27 | 92.06 | 42.69 |
| VG9_3 | 72769020 | 10988122020 | 70129580 | 10476014067 | 0.0263 | 97.59 | 92.82 | 42.47 |

| **TABLE 4** │ Comparison results of root sequences on the reference genome | | | | |
| --- | --- | --- | --- | --- |
| Sample Names | Total reads | Total mapped | Multiple mapped | Uniquely mapped |
| G9_1 | 79565184 | 76004054(95.52%) | 7324462(9.21%) | 68679592(86.32%) |
| G9_2 | 79232272 | 75762233(95.62%) | 7341926(9.27%) | 68420307(86.35%) |
| G9_3 | 86269428 | 82367509(95.48%) | 8160340(9.46%) | 74207169(86.02%) |
| V_1 | 80068028 | 76930726(96.08%) | 7783492(9.72%) | 69147234(86.36%) |
| V_2 | 84445126 | 81146557(96.09%) | 8004360(9.48%) | 73142197(86.62%) |
| V_3 | 69087002 | 66436268(96.16%) | 6437763(9.32%) | 59998505(86.84%) |
| VG9_1 | 71341098 | 68483224(95.99%) | 6070751(8.51%) | 62412473(87.48%) |
| VG9_2 | 70879396 | 67819406(95.68%) | 6158269(8.69%) | 61661137(86.99%) |
| VG9_3 | 70129580 | 67172688(95.78%) | 5989424(8.54%) | 61183264(87.24%) |

| **TABLE 5** │ Analysis of Photosynthetic Index of Parents and Hybrid | | | | |
| --- | --- | --- | --- | --- |
| Genetype | Pn(μmol*m^-2^*S^-1^) | Ci(μmol*m^-2^*S^-1^) | Gs(μmol*mmol^-1^) | Tr(μmol*mmol^-1^) |
| Va116 | 19.57b | 340.27b | 1.29a | 6.23b |
| GDH94 | 16.6c | 334.67c | 0.96b | 6.48a |
| Va116×GDH94 | 20.95a | 354.33a | 0.94b | 5.28c |
